# The fibroblast activation protein alpha as a biomarker of pulmonary fibrosis

**DOI:** 10.3389/fmed.2024.1393778

**Published:** 2024-09-19

**Authors:** Philomène Lavis, Ani Garabet, Alessandra Kupper Cardozo, Benjamin Bondue

**Affiliations:** ^1^Department of Pathology, Hôpital universitaire de Bruxelles, Université libre de Bruxelles, Brussels, Belgium; ^2^IRIBHM, Université libre de Bruxelles, Brussels, Belgium; ^3^Inflammation and Cell Death Signalling Group, Signal Transduction and Metabolism Laboratory, Université libre de Bruxelles, Brussels, Belgium; ^4^Department of Pneumology, Hôpital universitaire de Bruxelles, Université libre de Bruxelles, Brussels, Belgium; ^5^European Reference Network for Rare Pulmonary Diseases (ERN-LUNG), Frankfurt, Germany

**Keywords:** FAP, fibroblast activation protein, FAPI, PPF, IPF, progression, fibrosis, biomarker

## Abstract

Idiopathic pulmonary fibrosis (IPF) is a rare, chronic, and progressive interstitial lung disease with an average survival of approximately 3 years. The evolution of IPF is unpredictable, with some patients presenting a relatively stable condition with limited progression over time, whereas others deteriorate rapidly. In addition to IPF, other interstitial lung diseases can lead to pulmonary fibrosis, and up to a third have a progressive phenotype with the same prognosis as IPF. Clinical, biological, and radiological risk factors of progression were identified, but no specific biomarkers of fibrogenesis are currently available. A recent interest in the fibroblast activation protein alpha (FAPα) has emerged. FAPα is a transmembrane serine protease with extracellular activity. It can also be found in a soluble form, also named anti-plasmin cleaving enzyme (APCE). FAPα is specifically expressed by activated fibroblasts, and quinoline-based specific inhibitors (FAPI) were developed, allowing us to visualize its distribution *in vivo* by imaging techniques. In this review, we discuss the use of FAPα as a useful biomarker for the progression of lung fibrosis, by both its assessment in human fluids and/or its detection by imaging techniques and immunohistochemistry.

## 1 Introduction

Idiopathic pulmonary fibrosis (IPF) is a chronic, progressive interstitial lung disease (ILD) with an average survival of approximately 3 years (1). The only drugs available, nintedanib and pirfenidone, slow the progression of the disease, but the patient will still progress to respiratory failure. Lung transplantation is the only possible cure for IPF patients (2). In addition to IPF, there are many other diseases associated with the development of pulmonary fibrosis, such as hypersensitivity pneumonitis, occupational pneumonitis, connective tissue diseases, or idiopathic non-specific interstitial pneumonia. A progressive phenotype can also be present in these conditions, and accordingly, it has been shown that 18–32% of non-IPF ILD progressed despite an appropriate treatment (3–6) with a similar prognosis as IPF patients and a median survival of 3.7 years (7–9). Progressive pulmonary fibrosis (PPF) is defined by the presence of two of the three following criteria: (1) an absolute decline of more than 5% of forced vital capacity (FVC) or 10% absolute decline of the diffusion capacity of the lungs for carbon monoxide (DLCO) in 12 months; (2) worsening of the symptoms; (3) worsening of the fibrotic features on high-resolution computed tomography (HRCT) (10). PPF patients treated with nintedanib had a reduced decline of FVC compared to patients receiving placebo (9), indicating the necessity to identify markers of disease progression to better guide doctors in the choice of treatment.

Hypersensitivity and occupational pneumonitis, stage IV sarcoidosis, and ILD related to rheumatoid arthritis and systemic sclerosis are the main causes of PPF with an identified etiology (10, 11). In addition to the etiology of ILD, other risk factors were identified, particularly the presence of a usual interstitial pneumonia pattern (UIP) on the HRCT, the severity score of HRCT, and lower basal values of DLCO, FVC, or saturation of peripheral oxygen (5, 12–15). In progressor patients, survival is also influenced by age and sex, with older men being at greater risk of death (8). Moreover, each disease has associated risk factors for progression. For example, patients with rheumatoid arthritis are more likely to develop an ILD if they are obese, active smokers, or have an active disease (16). Regarding patients with ILD associated with systemic sclerosis, black ethnicity and the presence of anti-topoisomerase I (SCL-70) antibodies are risk factors of severity (17, 18).

In addition to those risk factors which are population-based factors, in clinical practice, there is an urgent need to find reliable patient-specific markers of fibrogenesis progression in order to guide clinicians in their choice of treatment, to determine the individual prognosis of each patient and the disease activity at a specific time (personalized medicine). Different blood biomarkers have already been studied in patients with IPF to assess their risk of progression or their survival (Table 1), but none is currently recommended for daily clinical practice, and even less so for PPF (2, 10). In the present article, we review different studies that reveal fibroblast activation protein α (FAPα) as a potential novel marker of fibrogenesis progression in fibrotic lung diseases.

**Table 1:** Summary of the potential blood biomarkers evaluated in the literature regarding progression and survival in patients with idiopathic pulmonary fibrosis (IPF).

Studied biomarker	Article	Aim of the biomarker	Cohort	Results
CA-125	Adegunsoye et al. (87)	Survival	240 IPF: 172 without treatment and 68 with antifibrotic Multicentric Plasma	Patients with antifibrotic treatment: worse survival in patients with higher levels of CA-125
	Maher et al. (88)	Progression and survival	Derivation cohort: 106 IPF and 50 controls Validation cohort: 206 IPF Multicentric Serum	Higher baseline levels in progressor IPF patients than stable IPF patients Increased levels of during 3-month follow-up in progressor patients Higher baseline levels and increased levels in deceased IPF patients than survivors
CXCL13	Adegunsoye et al. (87)	Survival	240 IPF: 172 without treatment and 68 with antifibrotic Multicentric Plasma	Worse survival in patients with higher levels of CXCL13, independent of antifibrotic treatment
	Guo et al. (89)	Progression and survival	126 IPF Monocentric Serum	Negative correlation between CXCL13 levels and decrease in FVC and DLCO Strong correlation between CXCL13 levels and worsening of HRCT lesions (reticulations, volume of ILD, and honeycombing) Better survival in patients with CXCL13 levels lower than 62 pg/mL
CCL18	Hamai et al. (90)	Diagnosis and survival	65 IPF, 31 bacterial pneumonias, 101 controls Monocentric Serum	Higher levels of CCL18 in IPF patients than controls. Discriminatory ability of levels higher than 38.7 ng/mL: sensitivity 66.2% and specificity 67.4% No prediction of 5-year mortality
	Raghu et al. (91)	Progression	124 IPF, 57 controls Multicenter Serum	No difference in CCL18 baseline levels between stable and progressor IPF patients A cutoff value of baseline 150 ng/mL: 48% of progressor in patients with higher baseline levels and 25% of progressor in patients with lower baseline levels
KL-6	Aloiso et al. (92)	Exacerbation and survival	Meta-analysis of 14 studies	Higher levels of KL-6 associated with a higher risk of exacerbation No association between KL-6 levels and survival
	Bennet et al. (93)	Progression	30 IPF, 30 NSIP, 14 controls Monocentric BALF	Higher levels of KL-6 in IPF and NSIP patients than controls but no difference between IPF and NSIP patients IPF patients: positive correlation between KL-6 levels and basal FiO_2_ and negative correlation with distance of 6-min walking test NSIP patients: negative correlations between KL-6 levels and FVC baseline, a distance of 6-min walking test, and final SpO_2_
	Bergantini et al. (94)	Progression	23 IPF Monocentric Serum	Strong correlation between KL-6 levels and DLCO variations but no correlation with FVC variation Decreased levels of KL-6 in patients treated with nintedanib
	Collard et al. (95)	Exacerbation and survival	67 IPF, 20 acute lung injury Monocentric Plasma	Higher levels of KL-6 in IPF patients with acute exacerbation than stable IPF patients and patients with acute lung injury No association between KL-6 levels and survival
	Guo et al. (89)	Progression and survival	126 IPF Monocentric Serum	Negative correlation between KL-6 levels and decrease in FVC and DLCO Strong correlation between KL-6 levels and worsening of HRCT lesions (reticulations, volume of ILD, and honeycombing) Better survival in patients with KL-6 levels lower than 800 U/mL
	Hamai et al. (90)	Diagnosis and survival	65 IPF, 31 bacterial pneumonias, 101 controls Monocentric Serum	Higher levels of KL-6 in IPF patients than controls and patients with bacterial pneumonia. Discriminatory ability of levels higher than 476 U/mL: sensitivity of 96.9% and specificity of 98.5% Independent risk factor of 5-year mortality
	Ikeda et al. (96)	Progression and survival	60 IPF patients Monocentric Serum	No prediction of progression by KL-6 levels
	Ikeda et al. (97)	Progression	163 IPF with antifibrotic treatment and 104 without Multicentric Serum	No prediction of progression by KL-6 levels
	Jiang et al. (98)	Progression	85 ILD with 20 IPF and 20 controls Monocentric Serum	Higher baseline levels in ILD patients than controls, in patients with FVC lower than 50% and in progressor than stable patients but no prediction of progression based on baseline KL-6 levels Prediction of progression by increased levels of KL-6 with a sensitivity of 86.4% and a specificity of 41.7% Increased levels of KL-6 are an independent risk factor for progression
	Raghu et al. (91)	Progression	124 IPF, 57 controls Multicenter Serum	No difference in KL-6 baseline levels between stable and progressor IPF patients
	Song et al. (99)	Progression and survival	118 IPF Monocentric Plasma	Higher levels in deceased patients than survivors KL-6 levels are not an independent risk factor of mortality
	Wakamatsu et al. (100)	Progression and survival	66 IPF Monocentric Serum	Higher levels of KL-6 in patients with deterioration of respiratory function Better prognosis of patients with KL-6 levels lower than 1,000 U/mL and stable than patients with KL-6 levels higher than 1,000 U/mL and rising
	Yoshikawa et al. (101)	Survival	49 IPF Monocentric Serum	Decreased KL-6 levels in stable patients and a tendency for an increase in progressor patients during a 3-month and 6-month follow-up No correlation between variations of KL-6 and FVC but a moderate negative correlation between variations of KL-6 and DLCO
	Yokoyama et al. (102)	Survival	27 IPF Multicentric Serum	Prediction of the risk of mortality by KL-6 levels higher than 1,000 U/mL with a sensitivity of 90% and a specificity of 70.6% KL-6 levels higher than 1,000 U/mL are an independent risk factor of mortality
MMP7	Adegunsoye et al. (87)	Survival	240 IPF: 172 without treatment and 68 with antifibrotic Multicentric Plasma	Patients without antifibrotic treatment: worse survival in patients with higher baseline levels of MMP7 Patients with antifibrotic treatment: worse survival if rising levels of MMP7 No decrease in MMP7 levels after antifibrotic treatment initiation
	Bauer et al. (103)	Progression	347 IPF, 100 controls Multicentric Serum	Higher levels in IPF patients than controls Negative correlation between MMP7 levels and FVC: lower FVC decline in patients with low baseline levels of MMP7 and higher FVC decline in patients with increased levels of MMP7 or higher baseline levels
	Hamai et al. (90)	Diagnosis and survival	65 IPF, 31 bacterial pneumonias, 101 controls Monocentric Serum	Higher levels of MMP7 in IPF patients than controls and patients with bacterial pneumonia. Discriminatory ability of levels higher than 5.56 ng/mL: sensitivity of 87.7% and specificity of 93.2% Independent risk factor of 5-year mortality
	Maher et al. (88)	Progression and survival	Derivation cohort: 106 IPF and 50 controls Validation cohort: 206 IPF Multicentric Serum	No prediction of progression by baseline levels or increased levels Higher risk of mortality in patients with higher levels of MMP7 but no association between higher mortality and rising levels of MMP7
	Raghu et al. (91)	Progression	124 IPF, 57 controls Multicenter Serum	No difference in MMP7 baseline levels between stable and progressor IPF patients
	Song et al. (99)	Progression and survival	118 IPF Monocentric Plasma	Higher levels of MMP7 in deceased IPF patients than survivors Prediction of the risk of mortality by MMP7 levels higher than 12.1 ng/mL with a sensitivity of 71% and a specificity of 54% MMP7 levels higher than 12.1 ng/mL are an independent risk factor of mortality The association of high levels of MMP7 and SP-A associated with the risk of progression: 42% of patients with high levels of both biomarkers will progress (decrease of more than 10% of FVC), and only 9% of patients with low levels of both biomarkers will progress
OPN	Adegunsoye et al. (87)	Survival	240 IPF: 172 without treatment and 68 with antifibrotic Multicentric Plasma	Patients without antifibrotic treatment: worse survival in patients with higher OPN baseline levels Patients with antifibrotic treatment: worse survival if rising levels of OPN Decrease levels of OPN 1 year after antifibrotic treatment initiation
	Gui et al. (104)	Exacerbation and survival	71 IPF, 20 controls Monocentric Serum	Higher levels of OPN in IPF patients with acute exacerbation than stable IPF patients and controls and higher levels in stable IPF patients than controls No correlation between OPN levels and FVC and DLCO Prediction of the risk of mortality by OPN levels higher than 3.24 ng/mL with a sensitivity of 57.1% and a specificity of 77.1% High levels of OPN are an independent risk factor for mortality
SP-A and SP-D	Adegunsoye et al. (87)	Survival	240 IPF: 172 without treatment and 68 with antifibrotic Multicentric Plasma	Patients without antifibrotic treatment: worse survival in patients with higher SP-D baseline levels Decrease in SP-D levels 1 year after antifibrotic treatment initiation
	Collard et al. (95)	Exacerbation and survival	67 IPF, 20 acute lung injury Monocentric Plasma	Higher levels of SP-D in IPF patients with acute exacerbation than stable IPF patients and patients with acute lung injury No association between SP-D levels and survival
	Hamai et al. (90)	Diagnosis and survival	65 IPF, 31 bacterial pneumonias, 101 controls Monocentric Serum	Higher levels of SP-A and SP-D in IPF patients than controls and higher levels of SP-D in IPF patients than patients with bacterial pneumonia Discriminatory ability of SP-A levels higher than 44 ng/mL: sensitivity of 66.2% and specificity of 76.5% Discriminatory ability of SP-D levels higher than 107 ng/mL: sensitivity of 84.6% and specificity of 88.6%
	Ikeda et al. (96)	Progression and survival	60 IPF patients Monocentric Serum	High levels of SP-A and SP-D predict progression and mortality at one-year follow-up but only SP-D levels are an independent risk factor for progression and mortality
	Ikeda et al. (97)	Progression	163 IPF with antifibrotic treatment and 104 without Multicentric Serum	High levels of SP-D are an independent risk factor for progression in IPF patients treated with pirfenidone but not in untreated IPF patients No prediction of progression by SP-A levels
	Kinder et al. (105)	Survival	82 IPF patients Monocentric Serum	Higher levels of SP-A associated with 1-year mortality
	Maher et al. (88)	Progression and survival	Derivation cohort: 106 IPF and 50 controls Validation cohort: 206 IPF Multicentric Serum	Higher levels of SP-D in progressor patients than stable but low variation over time Higher levels of SP-D associated with mortality but not increased levels of SP-D
	Raghu et al. (91)	Progression	124 IPF, 57 controls Multicenter Serum	No difference in SP-A and SP-D baseline levels between stable and progressor IPF patients
	Song et al. (99)	Progression and survival	118 IPF Monocentric Plasma	No difference in SP-A and SP-D levels in deceased IPF patients compared to survivors Prediction of the risk of mortality by SP-A levels higher than 80.3 ng/mL with a sensitivity of 75% and a specificity of 67.1% but not an independent risk factor of mortality The association of high levels of MMP7 and SP-A associated with the risk of progression: 42% of patients with high levels of both biomarkers will progress (decrease of more than 10% of FVC) and only 9% of patients with low levels of both biomarkers will progress
	Takahashi et al. (106)	Diagnosis and progression survival	52 IPF, 108 controls Monocentric Serum	Higher levels of SP-A and SP-D in IPF patients than controls Discriminatory ability of SP-A levels higher than 45 ng/mL: sensitivity of 78.8% and specificity of 94.4% Discriminatory ability of SP-D levels higher than 110 ng/mL: sensitivity of 84.6% and specificity of 95.4% Significant negative correlation between baseline levels of SP-D and decline in vital capacity but no association for SP-A levels Higher levels of SP-A and SP-D in deceased patients than survivors
	Wang et al. (107)	Survival	Meta-analysis of 21 articles	Higher levels of SP-A and SP-D associated with mortality
	Yoshikawa et al. (101)	Progression and survival	49 treated IPF patients Monocentric Serum	Decreased SP-A levels in stable patients and increased levels in progressor patients during a 3-month and 6-month follow-up. No variation of SP-D levels over time Negative correlations between variations of SP-A and SP-D and FVC and DLCO Prediction of survival by decreased levels of SP-A

BALF, bronchoalveolar lavage fluid; CA-125, cancer antigen 125; CA 19-9, cancer antigen 19-9; CCL18, CC-chemokine ligand 18; CXCL13, C-X-C motif chemokine 13; DLCO, diffusing capacity for carbon monoxide; FVC, forced vital capacity; ILD, interstitial lung disease; IPF, idiopathic pulmonary fibrosis; KL-6, Krebs von den Lungen-6; MMP7, matrix metalloproteinase 7; NSIP, non-specific interstitial pneumonia; OPN, osteopontin; SP-D, surfactant protein D; UIP, usual interstitial pneumonia.

## 2 The fibroblast activation protein α

**Figure 1:**
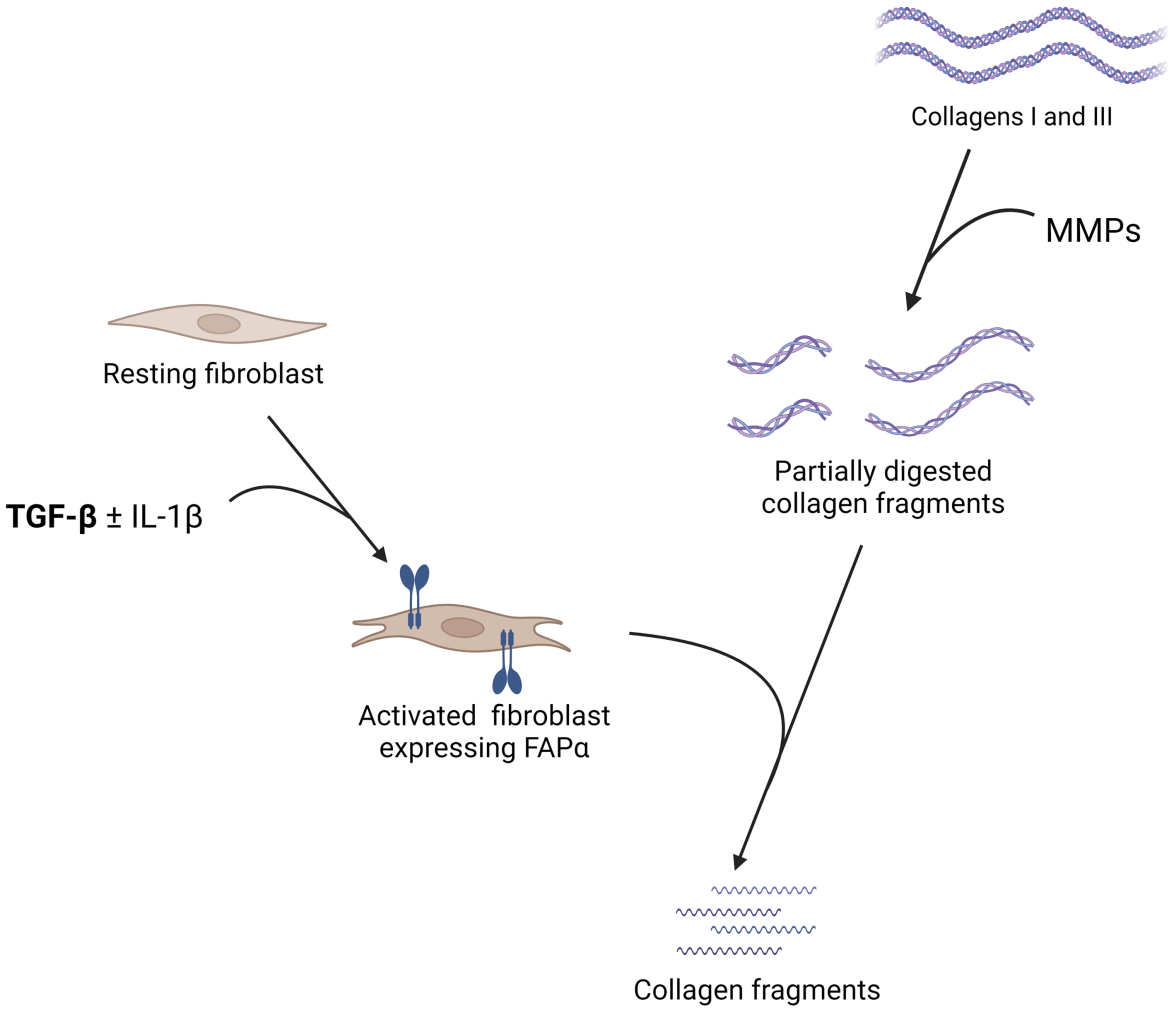
Summary of the pathophysiology of the fibroblast activation protein α (FAPα) in lung fibrosis. The expression of FAPα by fibroblasts is stimulated by transforming growth factor β (TGF-β) with a synergistic effect of interleukin 1β (IL-1β). FAPα is not able to cleave directly collagens I and III but complements matrix metalloproteinases (MMP) by cleaving partially digested collagen fragments. In the absence of FAPα, these partially digested collagen fragments accumulate in the lungs and more extensive fibrosis is observed. Created with Biorender.com.

FAPα is a 170 kDa transmembrane serine protease with extracellular activity. It is a member of the prolyl peptidases, along with dipeptidyl peptidase IV (DPPIV), with whom it shares 70% of amino acid sequence homology (19, 20). In addition to a dipeptidyl peptidase enzymatic activity, FAPα has its own endopeptidase enzymatic activity, also known as gelatinase (21, 22). Based on the enzymatic homology between FAPα and DPPIV, it was demonstrated that neuropeptide Y, brain natriuretic peptides, substance P, and peptide YY were FAPα substrates. However, it displayed no effect on chemokines cleaved by DPPIV (23). The dipeptidyl peptidase enzymatic activity of FAPα also inactivates fibroblast growth factor 21, through a cleavage of its C-terminal extremity (24, 25). Regarding its endopeptidase activity, FAPα has an antifibrotic activity as it complements matrix metalloproteinase 1 (MMP1) to cleave collagens I and III (Figure 1) but it is not able to cleave them alone. Of note, no enzymatic activity on collagen IV was detected (20, 26). Moreover, a soluble form of FAPα was identified, also named anti-plasmin cleaving enzyme (APCE) (27). APCE acts as FAPα and complements MMP1 to cleave collagens (26). In addition, APCE cleaves the N-terminal extremity of α2-anti-plasmin, generating a substrate that reduces blood clot degradation and delays fibrinolysis (28). Of note, human and murine FAPα share 89% amino acid sequence and have similar enzymatic activities (29).

FAPα is transiently expressed in certain fetal mesenchymal tissues but not in healthy adult tissues (30). Its expression is mainly found on fibroblasts from scar tissues, in pathological conditions of organ fibrosis and in the tumor microenvironment (30–34). FAPα contributes to tumor growth and metastasis through extracellular matrix degradation. Its expression is stimulated by transforming growth factor-β (TGF-β) with a synergistic effect of interleukin 1β (IL-1β) (35). In melanocytes and primary melanoma cells, FAPα expression was stimulated by ultraviolet radiation (36), whereas in an ovarian cancer cell line, FAPα induction was dependent on collagen I exposure (37). FAPα-expressing fibroblasts are different from myofibroblasts (expressing α-smooth muscle actin—αSMA) in their function; thus, FAPα-expressing fibroblasts tended to have a proteolytic profile, while αSMA-myofibroblasts were less involved in extracellular matrix remodeling but had contractibility properties (38).

## 3 FAPα and the physiopathology of lung fibrosis

It was previously demonstrated that FAPα was expressed in lesions of lung fibrosis, particularly in fibrotic interstitium and in fibroblast foci of IPF patients (39). Moreover, Wenlong et al. (40) were able to visualize lesions of fibrosis *in vivo* in mice by coupling FAPα with luciferase. They confirmed that FAPα was expressed by fibroblasts. However, its role in lung fibrogenesis is controversial; some studies show a pro-fibrotic role, whereas others show an antifibrotic one. Fan et al. (41) used two models to study lung fibrosis in FAPα-knock-out (FAP^KO^) mice: the bleomycin model and the thoracic irradiation model. The bleomycin model is the most widely used animal model to study lung fibrosis as it induces significant fibrosis in 3 weeks. During the first week, a strong immune response is observed, and lesions of fibrosis subsequently develop to reach a maximum at 21 days after the initiation of the model. However, after 28 days, the fibrosis tends to resolve (42–44). The thoracic irradiation model also induces a strong lung fibrosis that is similar to what is observed in humans after thoracic radiotherapy. The fibrosis takes, however, more time to develop between 24 and 30 weeks (43). In both models, Fan et al. (41) observed that wild-type (WT) mice had a better survival and developed less fibrosis than FAP^KO^ mice. In the basal state, FAP^KO^ mice had already more hydroxyproline lung content than WT mice. Moreover, they observed that isolated lung fibroblasts from FAP^KO^ mice exposed to TGF-β differentiated more into myofibroblasts. Finally, they observed that the FAP^KO^ mice presented an accumulation of collagen fragments in the lungs, confirming that FAPα cleaves products of MMP rather than complete collagen fibers (26). This antifibrotic role of FAPα was also observed by Kimura et al. (45) using the chronic bleomycin mouse model in two types of FAPα-deficient mice: FAP^KO^ mice and mice depleted of FAP^+^ cells. The chronic bleomycin model has the advantage of inducing an irreversible lung fibrosis, and the histological lesions are more similar to what is observed in IPF patients (46). These two mouse lines showed exacerbated lung fibrosis, and the FAP^KO^ mice a higher lung infiltration of immune cells (45). However, they did not observe any significant difference regarding fibrosis between transgenic and WT mice when lung fibrosis was induced by constitutive expression of active TGF-β. They hypothesized that the diverse fibrosis stimulus can trigger different subtypes of fibroblasts and therefore modulate the fibrotic response. Indeed, bleomycin could activate fibroblasts in FAP^+^ cells that present a proteolytic profile, whereas TGF-β would be more involved in fibroblast-to-myofibroblast differentiation and thus in tissue contraction (38, 45). On the contrary, Egger et al. (47) observed pro-fibrotic properties of FAPα. They induced fibrosis in WT mice with the chronic bleomycin model and treated them with an inhibitor of FAPα, namely, PT100. PT100-treated mice had a better survival and presented fewer areas of pulmonary fibrosis. It is to be noted that PT100 is an inhibitor of all the DPP family and not only FAPα (48). The contradictory results could therefore be explained by this lack of specificity, which is not found in mouse models using FAP^KO^ mice or mice depleted of FAP^+^ cells (41, 45).

Other inhibitors of FAPα were also studied in fibrosis affecting other organs. Yang et al. (49) performed a model of liver fibrosis and assessed an antifibrotic treatment with another quinoline-based FAPα inhibitor. Indeed, FAPα is expressed by hepatic stellate cells and activated fibroblasts in cirrhotic liver but not in healthy liver (31). The inhibitor of FAPα led to a lower mononuclear immune infiltrate, reduced collagen levels, and less fibrosis. As the proliferation of hepatocytes was higher, they hypothesized that the inhibitor also promoted regeneration of hepatocytes (49). Finally, Dienus et al. (32) studied fibroblasts from keloid scars and observed a higher expression of FAPα. The use of a FAPα inhibitor on isolated keloid fibroblasts reduced their invasion but did not influence procollagen I and fibronectin synthesis.

Overall, these studies showed contradictory results, with mice inactivated for FAPα appearing to be more susceptible to fibrosis (41, 45), whereas pharmacological inhibitors of FAPα seem to reduce the development of fibrosis in various mouse models (32, 47, 49). Therefore, more studies need to be performed to better understand FAPα pathophysiology as well as the precise mechanism of action of these inhibitors before considering it as a potential therapeutic target.

## 4 FAPα as a biomarker of fibrogenesis

Currently, there are no available biomarkers to identify fibrosis activity, particularly lung fibrogenesis. Various proteins were identified to be associated with a worse prognosis of IPF and therefore associated with the severity of the disease as cancer antigen 125, cancer antigen 19-9, or MMP7, but direct biomarkers of fibrogenesis are still missing (50). Moreover, the use of these biomarkers is not validated yet in daily clinical practice (10). FAPα is specifically expressed in fibroblast foci that are lesions of active fibrosis at the interface between healthy lung tissue and already fibrosed lung. Moreover, a soluble form of FAPα could be identified in biological liquids, rendering this protein a promising candidate as a biomarker for progressive lung fibrosis (21, 39). In the following paragraphs, we will discuss different studies that provide substantial evidence to support this assertion.

**Figure 2:**
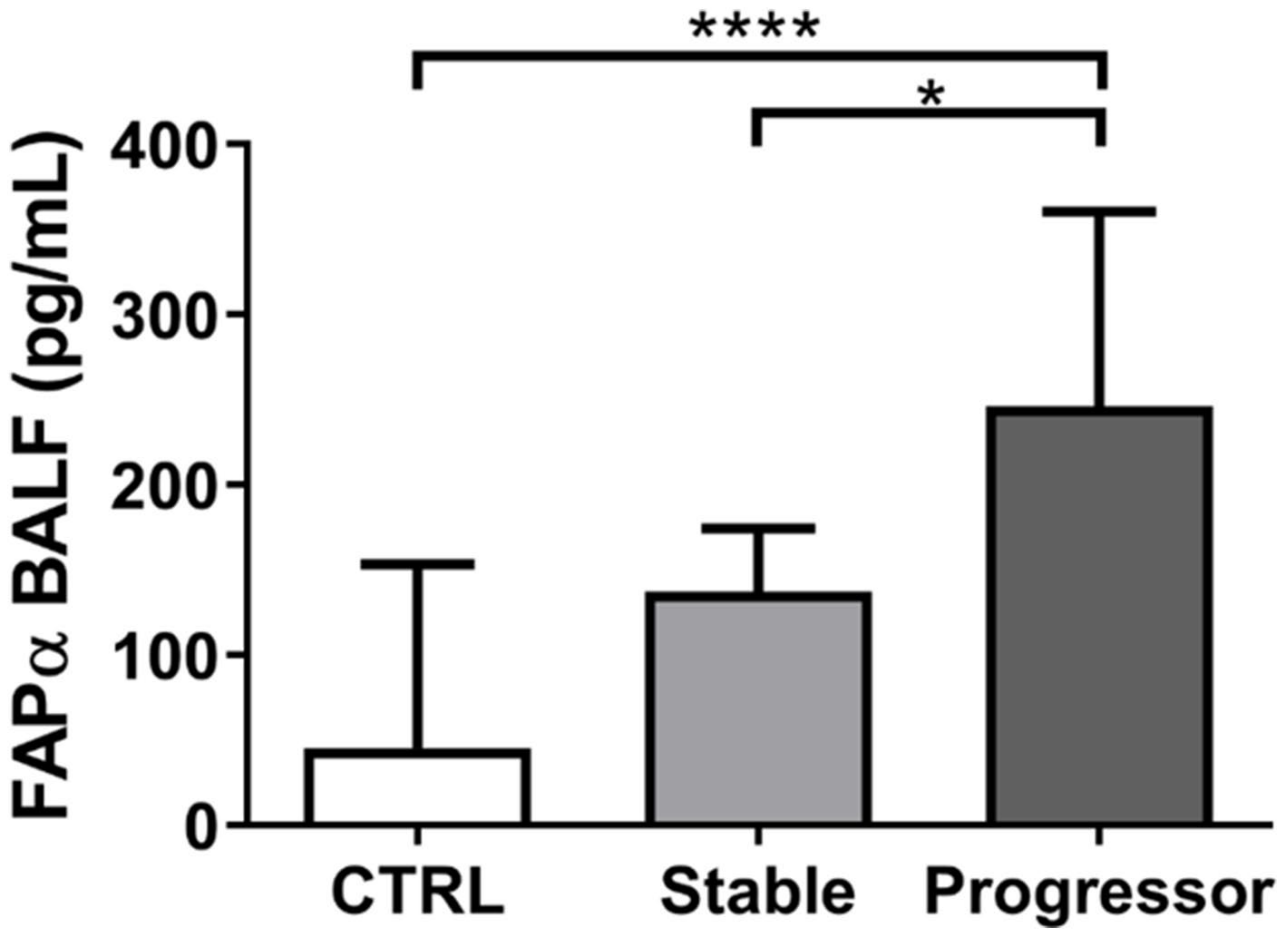
Assessment of FAPα levels in bronchoalveolar lavage fluid (BALF) from control subjects (CTRL) and patients with idiopathic pulmonary fibrosis (IPF) with either a stable or a progressive phenotype according to the 2022 ATS/ERS/JRS/ALAT Clinical Practice Guidelines (10). Data are reproduced from Lavis et al. (51).

Our group showed first that bleomycin-treated mice presented higher concentrations of FAPα in bronchoalveolar lavage fluid (BALF) as compared to control mice during the active phase of fibrogenesis. Importantly, a significant dose-response effect was observed. The levels of FAPα in BALF were correlated with weight loss and quantity of fibrosis. Moreover, a significant decrease in FAPα concentrations in BALF was observed in nintedanib-treated mice. Accordingly, IPF patients also showed higher levels of FAPα in BALF as compared to healthy controls. In addition, when IPF patients were classified into stable or progressors according to the 2022 ATS/ERS/JRS/ALAT Clinical Practice Guidelines (10), patients with a progressive disease had higher FAPα levels than controls and stable patients (Figure 2) (51). Finally, FAPα BALF levels higher than 192.5 pg/mL could predict the risk of progression, transplantation, or death with a sensitivity of 100% and a specificity of 90% (51). Of note, no association between FAPα concentrations with disease progression nor survival was found in a study analyzing the serum of 149 IPF patients (52), indicating local and not serum FAPα as a candidate biomarker for lung fibrosis. No comparison with control patients was performed (52).

Although promising, the data on BALF come from a single center and more studies using independent cohorts of patients should be analyzed to affirm FAPα as a biomarker of fibrotic lung diseases. Interestingly, other studies have also evaluated its use in other fibrotic diseases. Particularly, Uitte de Willige et al. (53) observed that FAPα plasma levels decreased in patients after transplantation for severe liver damage following hepatitis C infection. Lower plasma FAPα concentrations were associated with a lower fibrotic score in patients with non-alcoholic fatty liver disease and could also exclude severe fibrosis in patients with metabolic syndrome (54). Keane et al. (55) also showed that the activity of soluble FAPα was higher in cirrhotic patients. On the contrary, no significant difference was observed between patients with various atherosclerotic diseases and healthy controls. However, they observed that FAPα concentrations were higher in men than women and were associated with hyperlipidemia and body weight (56). Moreover, two independent groups did not highlight significant differences in blood FAPα levels in patients with systemic sclerosis, a disease characterized by a certain degree of skin fibrosis and involvement of other organs such as the lungs (57, 58).

In conclusion, currently, there are few studies evaluating the association of FAPα plasma or serum levels with fibrosis and the data are still controversial. On the contrary, preliminary data on the use of FAPα BALF concentrations as biomarker of lung fibrosis seem encouraging. As mentioned earlier, these results were obtained on a small cohort of IPF patients and multicenter studies should be performed to validate FAPα BALF levels as a biomarker of IPF and possibly as other progressive ILD.

## 5 FAPα inhibitors as markers of fibrogenesis

As previously described, FAPα is a marker of activated fibroblasts and cancer-associated fibroblasts. Based on specific enzymatic inhibitors of FAPα (FAPI), radiotracers were thus recently developed to identify *in vivo* the tumoral microenvironment. They are considered as pan-cancer radiotracers as PET/CT imaging of FAPI labeled with gallium-68 (^68^Ga) can specifically identify various prevalent cancers and their metastasis as breast, lung, colorectal, and prostate carcinoma (59). In addition to identifying cancer-associated fibroblasts, FAPI imaging seems also useful for non-oncological conditions, particularly some auto-immune diseases such as rheumatoid arthritis and Crohn’s disease but also fibrotic lesions in the heart and lungs (60–63). Particularly, ^68^Ga-FAPI was first tested in patients with lung cancer and lesions of lung fibrosis. Whereas the uptake of the radiotracer was identical between the tumor and the lesions of fibrosis, an early uptake of ^68^Ga-FAPI was observed in fibrotic lesions as compared to malignant ones (63). The uptake of ^68^Ga-FAPI in fibrotic lesions correlated with lung density (63) and was higher in IPF patients than controls (64). Bergmann et al. (65) evaluated the uptake of ^68^Ga-FAPI-04 in patients with systemic sclerosis-associated ILD and observed a heterogeneous uptake in the fibrotic lung, significantly higher than what was observed in controls. In this study, the highest uptake was observed in patients with extended ILD disease, greater impairment of respiratory tests, history of progression or progressive disease, or higher activity score of the disease. Moreover, they found a cutoff value of ^68^Ga-FAPI-04 uptake allowing them to predict the risk of progression. Finally, they noticed an association between the radiotracer uptake and the response to nintedanib, with an increased uptake in a patient with a worsening of its respiratory function, a status quo in two stable patients and a decreased uptake in two patients with an improvement of their respiratory function. The use of ^68^Ga-FAPI-04 was beneficial to evaluate myocardial fibrosis in patients with systemic sclerosis as the uptake was higher than patients without myocardial fibrosis and was associated with the risk of arrhythmia and heart failure (66). ^68^Ga-FAPI-46 was also able to identify lesions of pulmonary fibrosis following COVID-19 that were not detected by fluorodeoxyglucose coupled with fluor-18 (^18^F-FDG) (67). One study evaluated FAPI-74 coupled with ^18^F in IPF patients, where they observed that the uptake of the radiotracer was higher in IPF patients than the controls, and a strong correlation was observed between lung density and ^18^F-FAPI-74 uptake (64). Liu et al. (68) used single-photon emission computed tomography (SPECT) to detect FAPI by coupling it with ^99^Technetium. In agreement with the studies described above, a significantly higher uptake of FAPI was observed in the lower lobes of IPF patients than controls. SPECT imaging has advantage of being cheaper and delivering less radiation than PET/CT, therefore being a good alternative.

Preclinical murine models of lung fibrosis were also established to evaluate various FAPI-based radiotracers. Rosenkrans et al. (69) assessed ^68^Ga-FAPI-46 in the bleomycin lung model on days 7 and 14 after the instillation. They observed a higher and significant lung uptake of the radiotracer in bleomycin-treated mice than controls at these two timepoints, in contrast to lung density that was significantly different only at day 14. Compared with the uptake of ^18^F-FDG, less background in the heart and the brain were observed. Our group obtained similar results with ^18^F-FAPI-74; thus, a higher and significant uptake of the tracer was observed in bleomycin-treated mice than controls at days 10 and 16 after the instillation. We did not observe any significant difference at day 3, during the inflammatory phase nor at day 28 when the fibrosis is already established. Our results showed that ^18^F-FAPI-74 is a specific marker of fibrogenesis as lung uptake was strongly correlated with the development of fibrosis, assessed by lung density, lung content of hydroxyproline, and the Ashcroft modified scale. However, we could not observe significant differences in ^18^F-FAPI-74 uptake in mice treated with different doses of bleomycin that caused different levels of fibrosis (51).

FAPI uptake specificity for fibrogenesis was also observed in a model of tendinopathy with a high uptake of the tracer at day 7 after the injury and no significant difference after 4 weeks (70) and also allowed to discriminate inflammatory and fibrotic lesions in IgG_4_-related disease (71).

Song et al. (72) used a model of lung fibrosis induced by paraquat poisoning and observed a higher uptake of Al^18^F-NODA-FAPI-04 in poisoned mice than controls. Two patients after paraquat poisoning were also included, and a diffuse uptake in the lung bases was observed.

**Figure 3:**
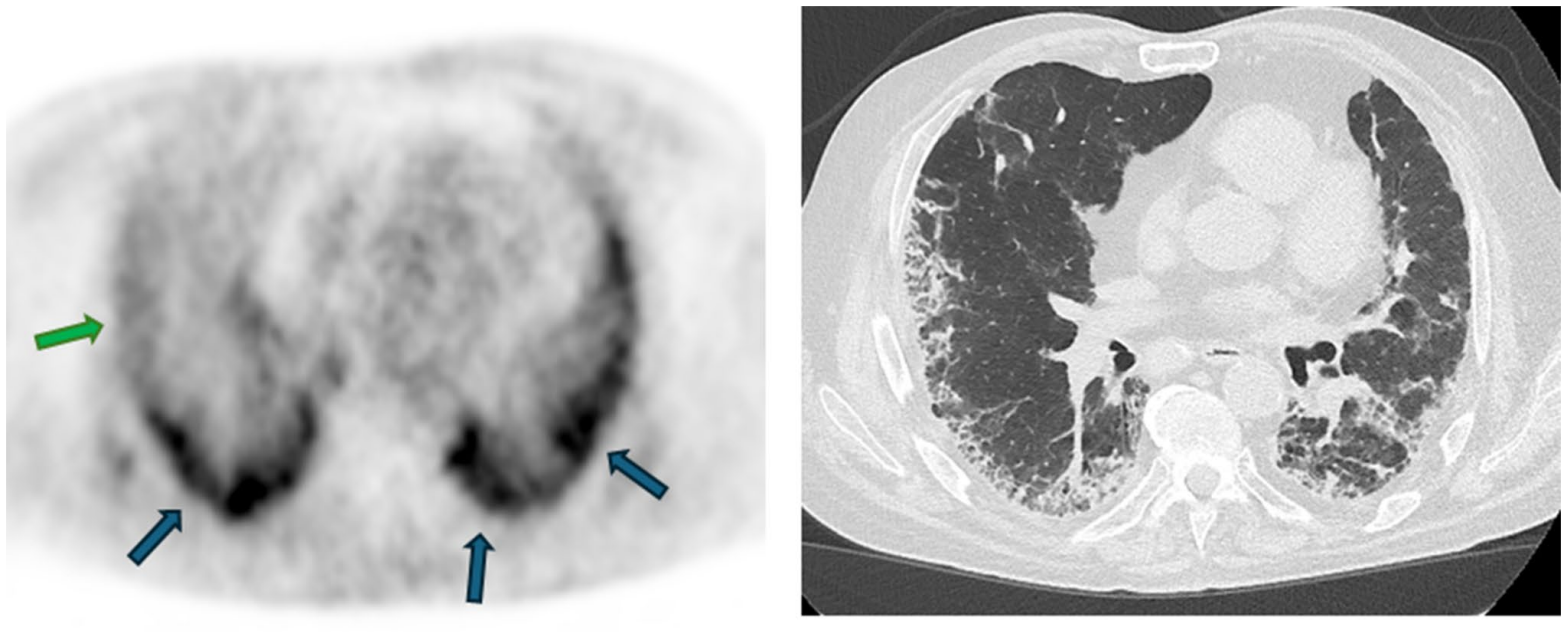
^68^Ga-FAPI uptake assessed by PET/CT in a patient with idiopathic pulmonary fibrosis and the corresponding high-resolution CT. An intense uptake of the radiotracer is observed in some subpleural lesions (blue arrows), whereas some present a weak uptake (green arrow), suggesting a lower fibrogenesis activity kindly provided by the Department of Nuclear Medicine of the Hôpital Universitaire de Bruxelles (HUB), Brussels, Belgium.

In summary, various studies showed that FAPI radiotracer uptake was higher in patients with lung fibrosis (IPF and non-IPF) as compared to controls, without being able to discriminate different etiologies of fibrosis. Compared to FDG, FAPI seems to be a promising specific marker of fibrogenesis that could help to differentiate active from inactive fibrotic lesions (Figure 3). Another limitation of ^18^F-FDG is that the intensity of the uptake was partly related to lung density, and this limitation is not found for FAPI radiotracers (73). Moreover, the response to antifibrotic treatments could not be assessed by ^18^F-FDG uptake (74), whereas encouraging preliminary results showed an association between the response to nintedanib and the uptake of FAPI in ILD patients with associated systemic sclerosis (65). ^68^Ga-DOTATATE, an analog of the somatostatin receptor was also previously assessed as a biomarker of lung fibrosis (75). However, contrary to FAPI, it is not entirely specific to fibroblasts and the detection of the somatostatin receptor 2a was found in alveolar macrophages, smooth muscle cells, epithelial bronchial cells, focally on endothelial cells and alveolar type 2 cells in bleomycin-treated mice (76). Moreover, a comparison between ^18^F-FDG uptake and ^68^Ga-DOTATATE was performed and showed that both presented the same distribution and uptake intensity (75). The assessment of ^129^Xenon red blood cell uptake by magnetic resonance enables pulmonary diffusion to be evaluated and thus reflects changes in lung microstructures (77, 78). However, it is not a direct marker of fibrogenesis. Recently, it was demonstrated that over a 12-month follow-up period, the tracer uptake was modified despite the absence of any significant decline in the pulmonary function tests. However, their cohort was quite heterogeneous with untreated patients and patients already receiving an antifibrotic treatment, making it impossible to assess whether ^129^Xenon uptake predicts response to antifibrotic treatment (78). As this imaging technique also seems promising, a study to compare FAPI and ^129^Xenon uptake to assess ILD progression and response to antifibrotic treatment could be performed.

Currently, FAPI coupled with ^68^Ga is the mostly used radiotracer (33, 63, 65), but some studies evaluating FAPI coupled with ^18^F or ^99^Tc have also demonstrated their feasibility and their ability to identify fibrotic lesions (64, 68). Further studies should be carried out to identify which pairing (subtype of FAPI and labeling) gives the best results in identifying lesions of fibrogenesis, with the lowest background and unspecific uptake. Overall, FAPI imaging is a promising tool to help clinicians determine the best timing to start antifibrotic treatments in non-IPF pulmonary fibrosis or evaluate the impact of other therapeutical interventions. We should keep in mind that even if the results are encouraging, more studies should be carried out to exactly determine how FAPI PET/CT imaging could be used as part of patient management.

## 6 FAPα expression as a marker of fibroblast foci

The identification of fibroblast foci on lung slides from patients with lung fibrosis is a hallmark of UIP (the pattern observed in IPF). In addition to UIP, some fibroblast foci can be observed in other conditions, such as non-specific interstitial pneumonia (NSIP). Fibroblast foci are found at the interface between healthy and fibrotic lung tissue, thus representing the active pulmonary fibrotic lesion (79). However, the origin of fibroblasts present in fibroblast foci is a matter of debate. One hypothesis is that they originated from alveolar epithelial cells undergoing epithelial-to-mesenchymal transition (EMT) under the influence of TGF-β (80). Another research group suggested that type 2 alveolar epithelial cells that had transited to a mesenchymal phenotype were not directly the main source of fibroblasts and myofibroblasts but promoted a pro-fibrotic environment leading to more fibroblast-to-myofibroblast differentiation (81).

The majority of the studies tend to show that the number and size of fibroblast foci are associated with poorer survival in patients with lung fibrosis (82, 83). Accordingly, the number of fibroblast foci was higher in patients with UIP than patients with NSIP in which the prognosis is better than in UIP (82). Collard et al. (84) observed a correlation between the number of fibroblast foci and a decrease in FVC and worsening of dyspnea at 6-month follow-up.

**Figure 4:**
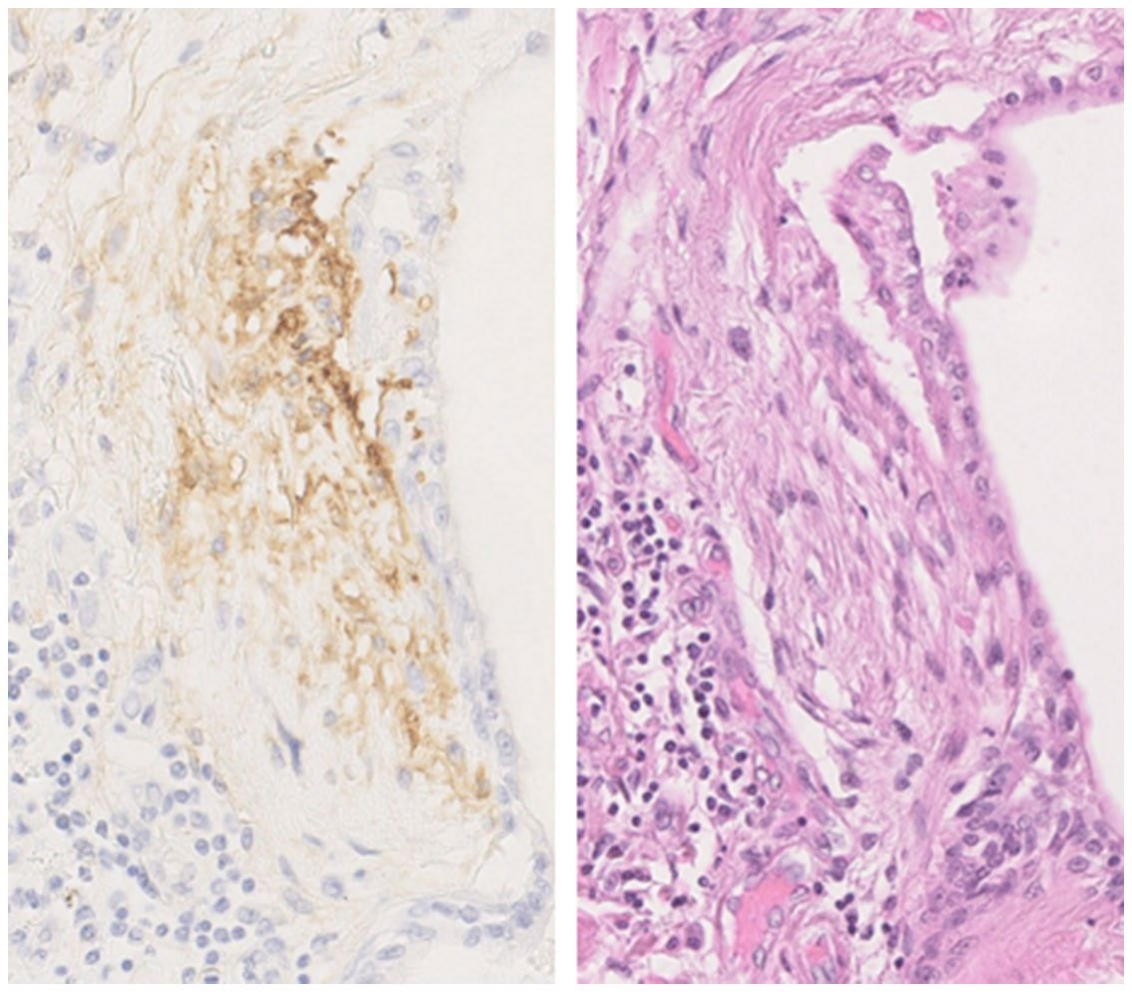
Fibroblast activation protein α (FAPα) immunostaining in a fibroblast foci from a lung explant of a patient with idiopathic pulmonary fibrosis with the corresponding hematoxylin-eosin staining. The use of FAPα immunostaining enables rapid identification of fibroblast foci and a more accurate assessment of their number. Magnification fold 200×. The images come from the Biobank of Pneumology—HUB. The protocol for FAPα immunostaining was detailed in Lavis et al. (51).

FAPα was shown to be a sensitive and specific marker of activated fibroblasts, with an expression found in fibroblast foci and fibrotic interstitium of UIP lesions but not on lung slides from patients with emphysema or healthy subjects (33, 39). Acharya et al. (39) also observed that FAPα expression on lung slides allowed the identification of some fibroblast foci that were not visible on the hematoxylin-eosin slide. They also showed that FAPα is more specific to fibroblast foci, as αSMA, a marker of transition of fibroblast to myofibroblasts, is also expressed by smooth muscle cells and therefore detected in vascular interstitium. In line with these studies, we also observed a clear staining of FAPα in fibroblast foci of patients with IPF (Figure 4). As the number of fibroblast foci seems to be associated with the prognosis of patients with lung fibrosis, FAPα immunohistochemistry staining could help clarifying the prognosis of patients for whom a cryobiopsy or a surgical lung biopsy is necessary for diagnosis. We have recently observed immunohistochemistry staining of FAPα in some hyperplastic alveolar epithelial cells that may be undergoing EMT, a hypothesis that needs to be tested (51).

Overall, FAPα staining appears to be a sensible marker of fibroblast foci that could be implemented in routine practice to help identifying fibroblast foci and therefore precise patients’ prognosis. Some studies could also be conducted to confirm the expression of FAPα by alveolar epithelial cells and verify whether it can be a novel marker of cells undergoing EMT in lung fibrosis.

## 7 Discussion

**Figure 5:**
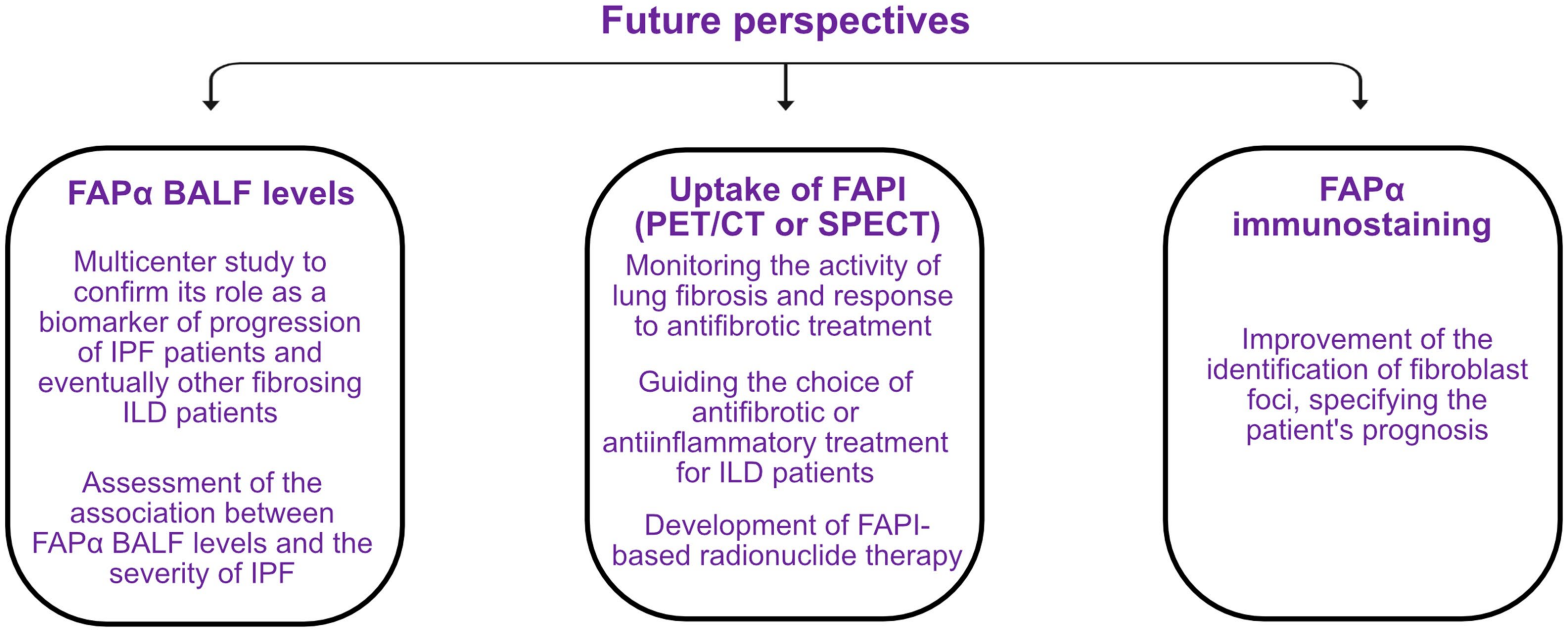
Perspectives on the use of the fibroblast activation protein α (FAPα) as a future biomarker of fibrogenesis in idiopathic pulmonary fibrosis (IPF) and other fibrosing interstitial lung diseases (ILD). BALF, bronchoalveolar lavage fluid; FAPI, FAPα inhibitor; PET/CT, positron emission tomography/computed tomography; SPECT, single-photon emission computed tomography.

Specific biomarkers allowing to identify IPF patients at risk of rapid progression at diagnosis or during the course of the disease are urgently needed. Moreover, a significant number of other lung fibrosis can evolve despite adequate treatment, and these biomarkers would be extremely helpful for these conditions. In this context, interest in FAPα has grown strongly in recent years, in particular due to its specificity for activated fibroblasts and the development of specific quinoline-based inhibitors that are able to target this protein and therefore visualize it by imaging techniques. In addition, the presence of a soluble form of FAPα, enabling the measurement of it in biological fluids, makes it a potential biomarker for the evaluation and follow-up of lung fibrosis. The perspectives regarding the use of FAPα as a biomarker of lung fibrogenesis are summarized in Figure 5.

Among fluids of interest, the assessment of FAPα in BALF seems particularly promising. Indeed, FAPα BALF levels are higher in IPF patients than controls and are associated with the progression of the disease (51). Currently, a BALF is performed in the majority of patients with a suspected ILD (2) and FAPα measurement could therefore be easily implemented as one of the parameters to be analyzed. Our study involved a limited number of IPF patients, and these results should be validated in larger multicenter studies but could eventually provide an assessment of a patient’s risk of progression at diagnosis. FAPα concentrations should also be measured on BALF from non-IPF ILD patients and could allow the identification of PPF patients in this group. The association between FAPα BALF levels and the severity of the disease could also be assessed.

For some ILD patients, the diagnosis of the etiology of lung fibrosis is difficult, and then, a surgical lung biopsy or a cryobiopsy is recommended (2). As FAPα helps identifying more precisely fibroblast foci and their number and size seem to be associated with the prognosis of the patient, this staining could be performed on lung samples and define the exact number of active lesions of the patient (39).

The detection of FAPI through the use of PET/CT or SPECT appears to be a powerful tool to identify active fibrogenesis, independent of the etiology of lung fibrosis. This could help monitor the activity of the disease, allowing to adapt drug doses to the patient and also assessing the response to antifibrotic treatment, as previously shown by Bergmann et al. (65). Compared to ^18^F-FDG, it is more specific for fibrogenesis, not influenced by lung density, and shows less background in brain and heart. Moreover, the uptake seems to be linked to active fibrotic processes, not inflammation (51, 69, 71). In certain ILD patients, this could help determine whether their pulmonary disease is predominantly inflammatory or fibrosing and thus help guide the choice of treatment. A future perspective on the use of FAPI is radionuclide therapy, directly targeting FAPα^+^ fibroblasts. Preliminary studies already assessed its efficacy in various advanced and progressive cancers, as FAPα is expressed by cancer-associated fibroblasts, and promising results were obtained (85). The treatment also seemed to be well tolerated (85, 86). Such studies on ILD patients could be considered given the specificity of FAPI for activated fibroblasts, mediating the progression of IPF and other PPF.

In brief, the identification of FAPα, whether in BALF, by nuclear imaging, or on lung sections, appears promising for assessing the risk of progression in fibrotic patients, response to treatment, and survival.
